# Assessing Patient Understanding and Satisfaction in Orthopedic Trauma and Elective Surgery Admissions

**DOI:** 10.7759/cureus.66924

**Published:** 2024-08-15

**Authors:** Shayan Zafar, Andrew P Dekker, Gur-Aziz Singh Sidhu, Adam T Stammer, Neil Ashwood

**Affiliations:** 1 Orthopedics and Traumatology, Barts and The London School of Medicine and Dentistry, London, GBR; 2 Trauma and Orthopedic Surgery, University Hospitals of Derby and Burton, Burton-on-Trent, GBR; 3 Trauma and Orthopedics, University Hospitals of Derby and Burton, Birmingham, GBR; 4 Orthopedic Surgery, Burton Hospital, Burton-on-Trent, GBR; 5 Trauma and Orthopedics, University Hospitals of Derby and Burton, Derby, GBR

**Keywords:** surgery, patient"s satisfaction, understanding, orthopaedics, clinic

## Abstract

Background

When seeking healthcare, patients often struggle to understand the information provided by healthcare professionals regarding their condition and treatment plan. Additionally, patient satisfaction with their experience can vary widely. Improved patient understanding and satisfaction are linked to better outcomes. This study aims to explore the factors influencing patient understanding to help healthcare professionals enhance these aspects.

Objective

This study evaluated the level of understanding and satisfaction among patients attending outpatient appointments. It also investigated factors influencing understanding by examining differences in results across various patient groups and analyzing these variations.

Methods

This study was conducted at Queens’ Hospital Burton, a level III trauma unit, over a three-week period in September 2023. Patients attending their orthopedic outpatient appointments were given a questionnaire, which included both bipolar 1-5 scale questions and open-ended text response questions.

Results

Patients generally reported high levels of understanding and satisfaction, averaging 90.34% and 96.20%, respectively. Those seen in a nurse-led clinic demonstrated significantly greater understanding of their condition compared to those seen by a physician (p = 0.0377). Additionally, trauma patients had a significantly higher level of understanding (p = 0.0167) and satisfaction (p = 0.0115).

Conclusions

To achieve better patient outcomes, it is crucial to optimize both patient understanding and satisfaction. Nurse-led clinics demonstrate higher levels of understanding, so identifying and incorporating the factors that contribute to this success into physician-led clinics is essential. These factors may include differences in communication methods, the resources provided, or the consultation setting. Additionally, the educational methods used with trauma patients appear more effective than those used for elective cases and should be evaluated to determine if they can enhance understanding and outcomes in other settings. Implementing evidence-based strategies for effective patient communication, such as maintaining good eye contact, avoiding medical jargon, and establishing rapport, could improve understanding and satisfaction and ultimately lead to better patient outcomes.

## Introduction

The relationship between patients and clinicians has undergone significant evolution over the past century. Increasing evidence suggests that a patient-centered approach, which addresses the holistic well-being of the patient, leads to better outcomes [1]. In orthopedics, as in other medical specialties, positive outcomes depend on various factors. While a surgeon’s experience and technical skills are crucial, a patient’s understanding of his or her diagnosis, treatment, and rehabilitation is arguably just as important. In orthopedics, although surgeons can realign or stabilize bones, it is the body’s healing process that ultimately resolves the issue and alleviates symptoms. Factors influencing this healing process, including lifestyle choices, risk management, and adherence to rehabilitation, are inherently patient-centered [2-4].

In addition to refining surgical techniques and practices, it is crucial for orthopedic healthcare providers to adopt a patient-centered approach to enhance outcomes. Consistent collaboration with patients through effective counseling on positive lifestyle factors, risk reduction, and adherence to rehabilitation regimes is essential. Evaluating patient understanding of this counseling can assess its effectiveness, as it has been associated with increased adherence, reduced non-attendance, and improved overall outcomes [5,6].

This study aimed to evaluate patients’ levels of understanding following trauma or elective surgery and assess the effectiveness of existing patient education methods. By identifying gaps in patient education, the study seeks to enhance understanding and, consequently, improve overall patient outcomes.

## Materials and methods

This study was conducted in the United Kingdom at Queens Hospital Burton, part of the University Hospitals of Derby and Burton NHS Foundation Trust. As an NHS hospital, it provides services free at the point of use and is classified as a level III trauma unit.

This cross-sectional study employed a patient questionnaire, chosen for its ease and speed, allowing for repetition after implementing interventions to evaluate their success. The questionnaire was collaboratively developed by the authors and follows the NHS guide to patient insight, Writing an Effective Questionnaire [7]. It was designed to be concise, use simple language, and include both easy-to-analyze closed questions with catch-all options and open-ended questions for more detailed feedback. A piloting phase was conducted to ensure reliability, resulting in adjustments to the question order and formatting for improved readability.

The questionnaire comprised nine main questions, with one question including two follow-up questions, totaling 11 questions. Initially, patients rated their understanding of their condition on a bipolar 1-5 scale. They were then invited to describe their condition in a free-text response, providing insight into how their perspectives might differ from those of clinicians. Next, patients were asked about their understanding of their treatment plan and whether they felt their questions were adequately addressed by the clinician. A subsequent free-text question sought to identify any additional information they desired, aiming to align the perceived importance of information with what clinicians consider essential.

The questionnaire also assessed patient satisfaction with their consultation and explored potential areas for service improvement. Finally, it included questions on information delivery strategies, evaluating the use of patient information leaflets, and gathering data on other sources of information utilized by patients.

Patients attending the fracture clinic and those awaiting elective surgery were randomly selected to participate in the study, with verbal consent obtained from each participant. Patients who were unable to complete the survey due to their illness or difficulties in understanding and retaining information were excluded. While patients under the age of 18 were included, the questionnaire was completed by their parents or guardians to maintain the study’s focus on adult understanding. Understanding pediatric patients, although important, was beyond the scope of this study. Patient-identifiable information was removed before analysis. Ethical approval was not required, as the study was classified as a service evaluation for a quality improvement project and deemed unnecessary by the local ethics committee.

Data were collected over three weeks, with 80 questionnaires distributed and 59 used for analysis. Of the remaining 21, 15 were not returned, and six were discarded due to incompleteness. The data from the completed questionnaires was analyzed with all identifiable information removed. Free-text responses were included to provide context for the closed questions.

The analysis examined various variables, including age, sex, condition type (elective or trauma), and clinic or surgical setting. Microsoft Excel (Microsoft Corporation, Redmond, Washington, United States) was used for data analysis. Unpaired t-tests were employed to compare different patient groups, such as those seen by nurses versus physicians, due to the unequal number of respondents in each group. A 95% CI was used to determine statistical significance. The study assumes that the respondents accurately represent the overall patient population.

## Results

**Figure 1:**
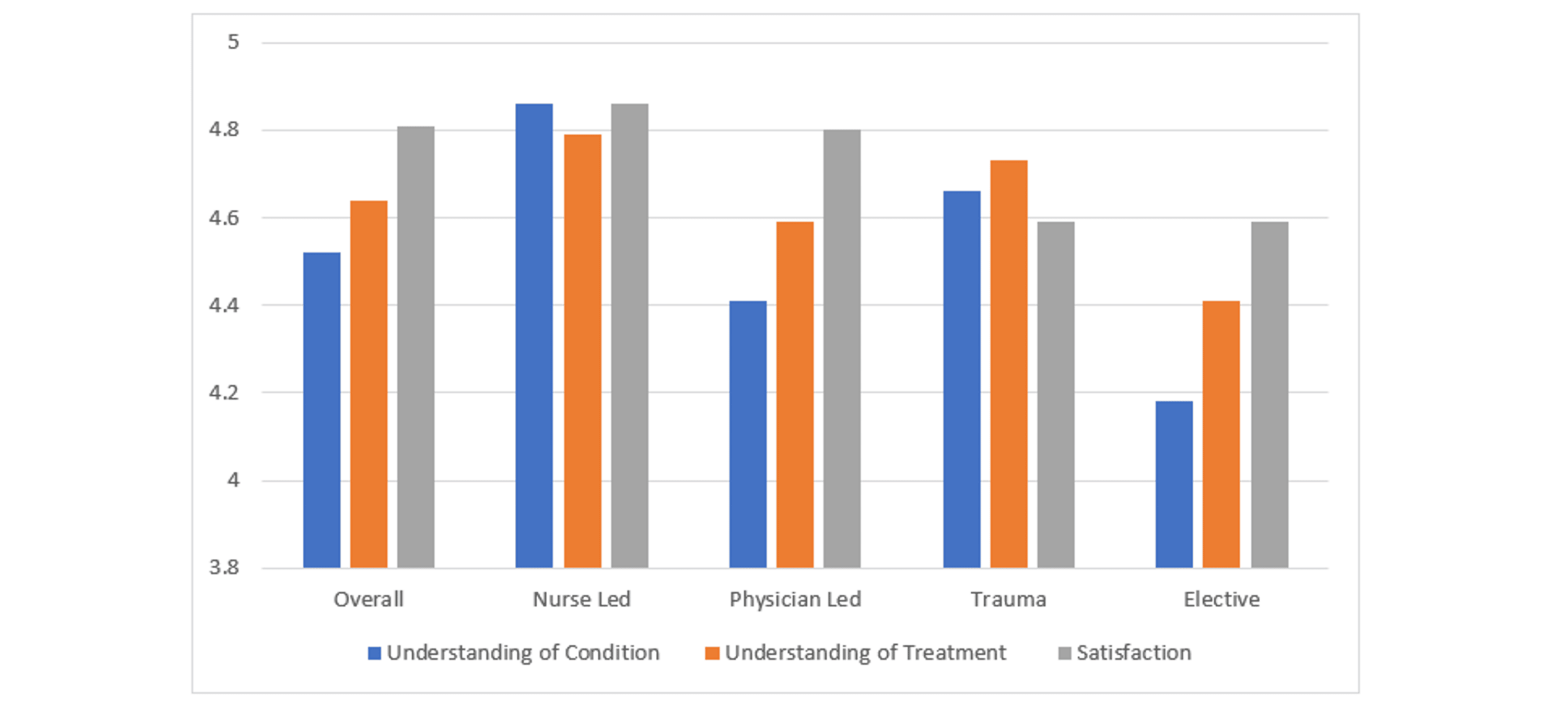
Levels of understanding and satisfaction across different patient groups

Overall, patients demonstrated high levels of understanding regarding both their condition and treatment plan, with very high satisfaction levels reported. On average, patients rated their understanding of their condition 4.52 out of 5, translating to 90.34%, and their understanding of their treatment plan 4.64 out of 5, or 92.76%. Overall satisfaction was rated 4.81 out of 5, equivalent to 96.20% (Figure 1).

When comparing respondents based on whether they were seen in a nurse-led clinic or by a physician, significant differences in understanding were observed. Patients seen by a nurse reported a higher average understanding of their condition (4.86) compared to those seen by a physician (4.41), with this difference being statistically significant (p = 0.0377) at a 95% CI. Although patients also reported a higher understanding of their treatment plan and greater overall satisfaction when seen by a nurse, these differences were not statistically significant (p = 0.3042 and p = 0.6500, respectively).

We also compared understanding and satisfaction levels between elective and trauma patients. Significant differences were found: trauma patients demonstrated a higher understanding of their condition, with an average rating of 4.66 compared to 4.18 for elective patients (p = 0.0167). Although trauma patients also reported a higher understanding of their treatment plan (4.73 vs. 4.41), this difference was marginally not statistically significant (p = 0.0699). Additionally, trauma patients reported significantly higher satisfaction levels, with an average rating of 4.90 compared to 4.59 for elective patients (p = 0.0115).

Responses were also analyzed by sex, comparing male and female patients. No statistical differences were found in any parameter. The average understanding of conditions for male and female patients was 4.50 and 4.53, respectively (p = 0.8776). For understanding the treatment plan, the averages were 4.75 for males and 4.56 for females (p = 0.2453). Satisfaction levels were reported at 4.79 for males and 4.82 for females (p = 0.7875).

Finally, patients were asked about the sources they used to educate themselves about their conditions and treatment plans. Thirty-seven percent of patients received a patient information leaflet, which received a usefulness score of 4.61 (92.22%). The most commonly reported source of information was the NHS website, used by 37.93% of respondents. Additionally, 29.31% of patients used Google for information, while 15% obtained information from family or friends.

## Discussion

Patient understanding is crucial for patient-centered care and significantly enhances outcomes [8]. Improved understanding leads to better adherence to treatment plans [9], which is vital for all specialties, particularly surgery and orthopedics. For both acute trauma and elective patients, a strong grasp of their condition and treatment plan increases adherence to preoperative, postoperative, and rehabilitation advice [10,11]. This adherence helps prevent treatment failures, enhances functional recovery, and results in better overall outcomes [10]. Therefore, monitoring and optimizing patient understanding are essential for improving patient outcomes.

Comparing the levels of understanding among different patient groups provides valuable insights into which groups have higher or lower levels of understanding and helps identify potential reasons for these differences. By analyzing these factors, targeted interventions can be developed for groups with lower levels of understanding. Implementing such interventions aims to enhance understanding, which in turn can improve overall patient outcomes.

This study revealed variations in levels of understanding between different patient groups, with significant differences observed based on whether patients were trauma or elective and whether they were seen by a nurse or a physician. Trauma patients exhibited a higher understanding of their condition compared to elective patients, but their understanding of the treatment plan was similar. This finding contrasts with other studies that have shown elective patients typically have better understanding levels [12,13].

One potential reason for this discrepancy might be the timing of data collection. Trauma patients often have less time to absorb information before their intervention, whereas elective patients might have months or years to comprehend their condition and treatment. The outpatient clinic setting of this study could have equalized this factor, as many elective patients presented before being listed for surgery. Another possible explanation is that the underlying conditions of elective patients, such as osteoarthritis or carpal tunnel syndrome, may be more complex than a straightforward bone fracture, potentially making them harder to understand. However, since the complexity of the treatment, particularly if surgery is involved, is comparable between trauma and elective cases, this may explain the lack of statistical differences in understanding of the treatment plan.

Nurse-led clinics have been recognized for improving patient outcomes, satisfaction, and access to care [14]. Specifically in orthopedics, they have demonstrated high patient satisfaction [15] and have been shown to optimize conditions for elective surgeries, thus reducing cancellations [16]. However, there is limited research comparing patient understanding between nurse-led and physician-led clinics.

This study found significant differences: nurse-led clinics were associated with higher levels of understanding of patients’ conditions compared to physician-led clinics, although understanding of treatment plans and levels of satisfaction were comparable between the two types of clinics.

Several factors could contribute to the observed differences in understanding. Feedback from patients in physician-led clinics frequently mentioned the complexity of the language used, which was less of a concern in nurse-led clinics. Additionally, 57.14% of patients in nurse-led clinics received a patient information leaflet, compared to 36.36% in physician-led clinics. Despite this disparity, the presence of leaflets did not significantly impact understanding or satisfaction.

Another potential factor is the clinic environment. Feedback indicated that physician-led clinics, often located in converted Nightingale-style rooms separated only by curtains, were noisy and distracting. In contrast, nurse-led clinics were held in quieter, separate rooms. Noise levels are a critical aspect of quality care delivery [17], affecting both patients and clinicians. This difference in environment may influence patient understanding and satisfaction and is particularly relevant in older hospital buildings across England.

The data for this study was collected from the orthopedics outpatient department at a single hospital during a specific study period, resulting in a relatively small sample size. Consequently, the conclusions drawn from this data require further validation through a multisite study to increase the number of responses and enhance reliability. The limited sample size is a notable limitation of this study, and a larger, multisite study conducted over an extended period could provide more robust and powerful data.

Additionally, this study may not fully represent the broader orthopedic patient population. As the study was conducted at a level III trauma unit, it primarily involved less complex acute trauma cases. Including patients from more specialized trauma centers could potentially influence the results and provide a more comprehensive understanding of patient experiences across different settings.

## Conclusions

Patient understanding and satisfaction are closely linked to improved outcomes, with higher levels of both being desirable. Understanding is notably higher among patients seen in nurse-led clinics and those with trauma compared to elective patients. Satisfaction also tends to be higher in these groups. To enhance understanding and satisfaction, interventions in patient communication, educational materials, and the consultation environment should be tested and evaluated. Implementing evidence-based communication strategies, such as maintaining good eye contact, avoiding medical jargon, and building rapport, could further improve understanding, satisfaction, and ultimately patient outcomes.

## Appendices

Clinic Satisfaction and Understanding Questionnaire

During clinical care, patients may receive a lot of information about their health and care. It is important that we communicate information in the best way possible so that patients get the most out of their care. We are interested in hearing your views in order to improve our services, and we kindly ask if you can take a moment of your time to fill out this patient questionnaire. All information in this questionnaire will be treated confidentially. If you have any questions, you can contact Shayan Zafar at shayan.zafar@nhs.net. Thank you in advance. 

_____________________________________________________________

Q1) How well do you understand your current orthopedic condition? 

(1 =Not at all, 5 = Completely) 

□ 1        □ 2        □ 3        □ 4        □ 5

Q2) How would you describe it?

..................................................................................................................................................................

Q3) How well do you understand your treatment plan? 

(1 =Not at all, 5 = Completely) 

□ 1        □ 2        □ 3        □ 4        □ 5

Q4) Did you feel like all of your questions were answered by the clinician?

□ Yes 

□ No

Q5) What, if any, further information would you like? ..................................................................................................................................................................

Q6) Overall, on a scale of 1 to 5 how satisfied are you with your consultation today? (1 being completely dissatisfied and 5 being completely satisfied)

□ 1        □ 2        □ 3        □ 4        □ 5

Q7) How can we improve? ..................................................................................................................................................................

Q8) Have you received a patient information leaflet?

□ Yes  □ No

Q8b) If yes, how useful was it? (1 =Not at all, 5 = Very useful)

□ 1        □ 2        □ 3        □ 4        □ 5        □ Did not read  

Q8c) How can it be improved?

..................................................................................................................................................................

Q9) What other sources of information have you used for your condition or treatment?

□ Google

□ NHS Website

□ Family/ Friends

□ Social Media

□ Other

Please specify 

..................................................................................................................................................................

**End of Questionnaire. Thank you**.
